# Therapeutic Drug Monitoring of Meropenem in Neonate with Necrotizing Enterocolitis: A Challenge

**DOI:** 10.1155/2016/6207487

**Published:** 2016-09-15

**Authors:** Steven De Keukeleire, Daniëlle Borrey, Wim Decaluwe, Marijke Reynders

**Affiliations:** ^1^Department of Laboratory Medicine, AZ Sint-Jan Bruges, Bruges, Belgium; ^2^Department of Pediatrics, Neonatal Intensive Care, AZ Sint-Jan Bruges, Bruges, Belgium

## Abstract

Necrotizing enterocolitis (NEC) continues to be a major cause of neonatal morbidity and mortality. We describe the added value of therapeutic drug monitoring by presenting the case of a preterm infant with severe NEC treated with meropenem. Dosing strategy will achieve adequate patient outcome when treating pathogens with elevated MIC. As safe as meropenem is, there are not enough data for 40 mg/kg, every 8 h infused over 4 h; accordingly, strict monitoring of blood levels is mandatory. Based on our findings, a 4 h prolonged infusion of 40 mg/kg meropenem, every 8 h, will achieve an adequate patient outcome.

## 1. Introduction

Necrotizing enterocolitis (NEC), one of the most common gastrointestinal emergencies in preterm infants, continues to be a major cause of neonatal morbidity and mortality. Early recognition and aggressive management have improved clinical outcome, although the exact etiology remains unknown. Given the polymicrobial intra-abdominal nature of this infection, broad-spectrum or combination antimicrobial agents are most often initiated [1]. Meropenem, because of its broad-spectrum activity, would be an agent of great utility [2]. Unfortunately, little information is available regarding the pharmacokinetic properties of meropenem in the neonatal patient population. Therefore, we want to highlight the importance of therapeutic drug monitoring (TDM) in the treatment of NEC by presenting this case.

## 2. Case Presentation

**Figure 1:**
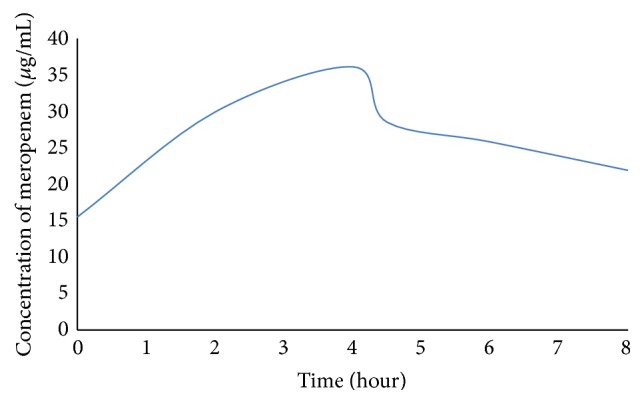
Typical serum concentration-time profile of meropenem given as a 4 h prolonged infusion of 40 mg/kg, every 8 hours.

A preterm male infant was born by cesarean section at 30 and 5/7 weeks' gestation weighing 1,140 grams, of which the monochorionic diamniotic twin pregnancy was complicated by twin-to-twin transfusion syndrome (grade I) and severe intrauterine growth restriction. Maternal antibiotics and betamethasone were administered prior to delivery. Apgar scores of 7 and 9 were assigned at 1 and 5 minutes of life. He knew a good start with a spontaneous heart activity and received 30% oxygen and PEEP by Neopuff, which was replaced by nasal continuous positive pressure ventilation (CPAP), which persisted during his stable transport to the neonatal intensive care unit (NICU) and continued until day 4. He was started on caffeine for prevention of apnea and bradycardia, and it could be stopped on day of life (DOL) 16. Due to immaturity of preterm intestinal mucosa and risk for development of NEC minimal enteral feeds were initiated on DOL 2 and only on DOL 7 the quantity was prudently increased, reaching full enteral feeds by DOL 17. On DOL 29 he had an acute onset of progressive abdominal distention and general malaise, clinical and radiologic compatible with NEC stage IIb (modified Bell's staging). Abdominal RX findings included explicit signs of pneumatosis intestinalis at the right hypochondrium and the left flank and residual air at the level of the vena porta bifurcation. Enteral feeds were stopped, and gastric decompression with continuous suctioning and a sepsis workup was initiated. A complete blood count, metabolic profile, blood gas, and blood culture were drawn, which revealed anemia (8.6 g/dL, normal range: 10.7–17.1 g/dL) and thrombocytopenia (26.10 E^9^/L, normal range: 150–450.10 E^9^/L). He was started on IV cefotaxime, vancomycin, and metronidazole, serial abdominal exams were performed, and the pediatric surgical team was consulted. Blood cultures remained negative. Seven days after initiation of medical therapy (DOL 36), the surgical team reevaluated the infant. It was decided to proceed with an exploratory laparotomy given the progressive clinical decline and respiratory alerts. Intraoperative findings included malodorous and purulent free fluid present in abdomen, clear necrotizing enterocolitis (NEC grade IIIb) affecting the whole colon (with 4 covered perforations) and the distal small bowel, with intra-abdominal leakage of feces and excessive bleeding leading to a complete colectomy until the sigmoid, while creating a jejunostomy and small fistula. On postoperative day 6, a focal fluid collection was detected by ultrasonography. Peritoneal fluid culture was positive for* Enterobacter cloacae* complex, while peripheral blood cultures remained negative. The antimicrobial therapy was changed to IV meropenem, amikacin, and fluconazole based on* E. cloacae* complex susceptibilities (Minimal Inhibitory Concentration (MIC): 1 *μ*g/mL (*E*-test)). Based on the severity of the situation, appropriate meropenem dosage was optimized by means of real-time TDM monitoring. Serum samples were analysed using a validated High-Performance Liquid Chromatography method with Diode-Array Detection. Meropenem was initially administered as a 30-minute infusion of 20 mg/kg, every 8 h. TDM monitoring, conducted 1 h after the infusion of the fourth dose, revealed the complete absence of meropenem. The optimal transport conditions (immediately after collection of the blood it was transported on ice and toxicological analysis began) were respected. Therefore the dose was increased to 30 mg/kg (Table 1). However based on the further clinical deterioration of the patient and the objectivation of fast metabolization of the antibiotic, the meropenem protocol was switched to a 4 h prolonged infusion of 40 mg/kg, every 8 h (Table 2 and Figure 1). The calculated elimination half-life was approximately 1.2 h. A 4 h prolonged infusion of 40 mg/kg meropenem, every 8 h, will achieve an adequate antibiotic exposure against* E. cloacae* complex with T > MIC: 50% of time > 4 *μ*g/mL and 75% of time > 2 *μ*g/mL (creatinine value: 0.29 mg/dL; normal range: 0.31–0.88 mg/dL). Based on the infected intra-abdominal collection and increase of inflammatory parameters (CRP: 122 mg/L; normal range: <5 mg/L) a new laparotomy was performed on DOL 52 for drainage of it. Intraoperatively the infected collection and the existing but necrotic jejunostomy were removed. The frailty of the bowel was too high to create a new stoma, so a first Petzer sonde was placed in the terminal ileum and sorted cutaneously, and a second Petzer sonde was placed for a more proximal perforation. In the light of the infectious problem, very difficult wound healing and complete dehiscence of the wound suture were observed, followed by slow granulation over the following weeks. Peritoneal fluid cultures remained positive for* E. cloacae* complex with a gradual increase of MIC until an intermediate susceptibility value, 6 *μ*g/mL (EUCAST), but with negative molecular carbapenemase producing Enterobacteriaceae screening. On DOL 59, after complete eradication of* E. cloacae* complex (repeated negative cultures), the antibiotic regimen was switched to IV ciprofloxacin and metronidazole for a 14-day course, after 18 days of extended meropenem infusion. A fourth laparotomy followed aiming to create a distal stoma, while during the same intervention the proximal stoma was closed. The patient received 5 weeks of broad-spectrum antibiotics in total. He was discharged in a stable clinical condition at 36 weeks of postmenstrual age weighing 4,370 grams.

## 3. Discussion

Severe NEC is a life-threatening condition requiring prompt intravenous therapy with broad-spectrum antimicrobial agents [1]. Meropenem, a time-dependent carbapenem antibiotic, with broad-spectrum activity, is active against a wide variety of Gram-negative and Gram-positive microorganisms and offers good penetration of body fluids and tissues. It has been shown to be well tolerated by children and neonates, including preterm babies, with the advantage of allowing monotherapy instead of combined therapy. Unfortunately, meropenem had not been approved for use in children younger than 3 months of age and thus dosage recommendations cannot be made for this age group. Meropenem dosing in young infants is often based on pharmacokinetic (PK) data extrapolated from adults or older children. For meropenem, activity is dependent on the percentage of the dosing interval with concentration above the minimum inhibitory concentration (%T > MIC) [3, 4]. Given the large interpatient variability, optimization of meropenem use in neonates may require TDM. Smith et al. in 2011 proposed different doses depending on gestational and postnatal age with a creatinine value of <1.7 mg/dL. Furthermore they suggest a target of 75% T > 2 *μ*g/mL (20 mg/kg, every 8 h) as premature infants could be thought of as immune compromised for whom a target of 40–50% T > MIC may be insufficient. These targets were defined based on the Clinical and Laboratory Standards Institute (CLSI) recommended MIC breakpoints of meropenem for* Pseudomonas aeruginosa* (susceptible: <2 *μ*g/mL and resistant: >4 *μ*g/mL) which closely correspond to the European Committee on Antimicrobial Susceptibility Testing (EUCAST) breakpoints (susceptible: ≤2 *μ*g/mL and resistant: >8 *μ*g/mL) [3]. Limited studies indicated a meropenem dosing strategy of 20 mg/kg every 8 h [2–5]. Moreover Van den Anker et al. in 2009 favored a 40 mg/kg dose to treat more resistant microorganisms (MIC > 4 *μ*g/mL) [2]. The selected dosing interval will have a major impact on the adequacy of the chosen dosing strategy. Studies have emphasized that a prolonged 4 h infusion may be beneficial for microorganisms with increased MIC [2, 4, 5]. The observed meropenem concentrations in our patient exceeded the MIC of* E. cloacae* complex (2 *μ*g/mL) and PK-PD target of 75% of the dosing interval > MIC with the assumption that neonates are immune-compromised host. In general, for intermediate or resistant microorganisms (with meropenem MICs ≥ 2 *μ*g/mL) better PK/PD target attainment is obtained with prolonged 4 h infusion [2, 4, 5]. Our finding that prolongation of meropenem infusion results in an advantageous PK/PD profile is consistent with the previous results reported [2, 4, 5]. The reason for the observed short half-life (1.2 h) and low initial meropenem concentration remains unclear. Nevertheless, it must be understood that physiologic changes may influence the PK of meropenem, especially in low birth weight neonates when recovering from septicemia [6]. A potential drawback is associated with the degradation of meropenem after reconstitution; however the degradation will be <10% over 12 h at a concentration of 4% at room temperature (<25°C) [7]. Our case confirms previous findings: meropenem is well tolerated and effective against multiresistant Gram-negative microorganisms and can be used safely in neonates [2, 8, 9]. In conclusion, TDM can be used as an additional tool for clinicians to optimize dosing and to improve clinical outcome, allowing a tailored therapy for each single patient. A 4 h prolonged infusion of 40 mg/kg meropenem, every 8 h, will achieve an adequate antibiotic regimen against* E. cloacae* complex with T > MIC: 50% of time > 4 *μ*g/mL and 75% of time > 2 *μ*g/mL.

## Figures and Tables

**Table 1:** Serum meropenem concentrations given as a 30-minute infusion of 30 mg/kg, every 8 hours.

Time (min)	Concentration of meropenem (*μ*g/mL)

0	54
10	32
20	28

**Table 2:** Serum meropenem concentrations given as a 4 h prolonged infusion of 40 mg/kg, every 8 hours.

Time (min)	Concentration of meropenem (*μ*g/mL)

0	15
120	30
240	36
270	29
360	25
480	22

## References

[1] Neu J., Walker W. A. (2011). Necrotizing enterocolitis. *The New England Journal of Medicine*.

[2] Van den Anker J. N., Pokorna P., Kinzig-Schippers M. (2009). Meropenem pharmacokinetics in the newborn. *Antimicrobial Agents and Chemotherapy*.

[3] Smith P. B., Cohen-Wolkowiez M., Castro L. M. (2011). Population pharmacokinetics of meropenem in plasma and cerebrospinal fluid of infants with suspected or complicated intra-abdominal infections. *The Pediatric Infectious Disease Journal*.

[4] Bradley J. S., Sauberan J. B., Ambrose P. G., Bhavnani S. M., Rasmussen M. R., Capparelli E. V. (2008). Meropenem pharmacokinetics, pharmacodynamics, and monte carlo simulation in the neonate. *The Pediatric Infectious Disease Journal*.

[5] Ohata Y., Tomita Y., Nakayama M., Kozuki T., Sunakawa K., Tanigawara Y. (2011). Optimal dosage regimen of meropenem for pediatric patients based on pharmacokinetic/pharmacodynamic considerations. *Drug Metabolism and Pharmacokinetics*.

[6] van Enk J. G., Touw D. J., Lafeber H. N. (2001). Pharmacokinetics of meropenem in preterm neonates. *Therapeutic Drug Monitoring*.

[7] Berthoin K., Le Duff C. S., Marchand-Brynaert J., Carryn S., Tulkens P. M. (2010). Stability of meropenem and doripenem solutions for administration by continuous infusion. *The Journal of Antimicrobial Chemotherapy*.

[8] Köksal N., Hacimustafaoğlu M., Bağci S., Çelebi S. (2001). Meropenem in neonatal severe infections due to multiresistant gram-negative bacteria. *Indian Journal of Pediatrics*.

[9] Cohen-Wolkowiez M., Poindexter B., Bidegain M. (2012). Safety and effectiviness of meropenem in infants with suspected or complicated intra-abdominal infections. *Clinical Infectious Diseases*.

